# Significance of Hoarseness and Aphonia in Cases of Pulmonary Phthisis

**Published:** 1888-03

**Authors:** Barclay J. Baron

**Affiliations:** Physician in Charge of the Throat Department of the Bristol General Hospital


### SIGNIFICANCE OF HOARSENESS AND APHONIA IN CASES OF PULMONARY PHTHISIS.

BY

BARCLAY J. BARON, M.B., EDIN.,

*Physician in Charge of the Throat Department of the Bristol General Hospital.*

---

IT is a well recognised fact that *hoarseness* and *aphonia* are, in a certain proportion of all cases of pulmonary phthisis, added to the usual symptoms of the latter disease.

Hoarseness is more commonly met with in lung disease than aphonia; in fact, the latter may be said to be decidedly rare.

Having had opportunities of examining a great many cases of lung consumption in which one or other of these symptoms was present, I have found that the pathological conditions underlying and causing them are very different in different cases. The pathological conditions varying widely, it necessarily follows that the diagnosis, prognosis, and treatment of the throat affection must vary equally widely; the great importance of an exact knowledge of the state of the larynx will be at once recognised, since it throws so much light on diagnosis and prognosis, and suggests accurate treatment of a curative or palliative character.

The conditions of the throat which I have found on laryngoscopic examination in cases of pulmonary phthisis, are as follows:

1. *Simple Catarrh of the Larynx.*—In this disease the mucous membrane of the larynx is reddened and swollen; the vocal cords are swollen, congested, and pink in colour; and there is hyper-secretion of mucus. We have to do, in fact, with a catarrhal laryngitis of greater or less severity. It is worthy of note that *aphonia* is more frequently observed in this condition than in almost any other, which is likely to be associated with pulmonary phthisis. I have seen cases of this throat trouble get quite well, and that just as rapidly when the lungs are affected, as when they are healthy; at the same time, I think that we ought to be guarded in our prognosis, and hesitate to pronounce such a catarrh simple, until we have allowed time to elapse, and have had the opportunity of seeing, after repeated examination, whether or not it is the beginning of a specific tubercular inflammation, which some such cases will undoubtedly prove to be. Careful, non-irritating and soothing measures of treatment, by inhalations, &c., ought to be adopted; and advice given as to the avoidance of cold damp weather, and irritating food and drink—also too much talking and all singing are injurious.

2. *Anæmia of the Larynx.*—Frequently, on looking at the fauces of a phthisical patient affected with hoarseness, we can see the extreme degree of anæmia that is present. The uvula, palate and pharynx all look bloodless and of one uniform white or drab colour, with scarcely a blood-vessel to be seen.

On examining the larynx we find exactly the same state of affairs as in the pharynx, the mucous membrane of the epiglottis and the whole of the larynx is white or drab, the vocal cords move sluggishly, and are unable to come tightly together on phonation, thus rendering clear voice impossible, and hoarseness, very rarely aphonia, results.

Now this anæmia is only a local expression of the general bloodlessness; but such a larynx is distinctly more liable to become infiltrated with tubercle in its sub-mucous coats, than one in which there is more healthy blood circulating. I know from experience that if we periodically examine such a larynx, we shall see in a certain proportion of cases, that the mucous membrane covering and surrounding the arytenoids becomes swollen, looks more opaque, and appears to have something deposited beneath it. This "something," which is tubercle, becomes gradually larger, and in fact constitutes the commencement of phthisis laryngea.

The treatment is mainly general, by blood tonics, &c., and avoidance of all hurtful influences which can act on the throat, such as damp and cold air, hot and peppery foods, &c. Careful periodical examination with the laryngoscope is also of prime importance.

3. *Paralysis of one Vocal Cord.*—I have examined a few cases of lung disease in which there was also present either a very considerable amount of hoarseness or of complete aphonia, the cause of which was immobility of one vocal cord on phonation. The appearance presented by the larynx is, in fact, exactly what one finds where a thoracic aneurism or other tumour is compressing one recurrent laryngeal nerve—*i.e.*, the affected vocal cord is usually in the cadaveric position, and does not move on phonation.

The causes of this condition are, either enlarged bronchial glands or induration with pleuritic thickening of the apex of the lung; and it is noticeable, that, whereas from anatomical reasons the left recurrent laryngeal nerve is the one that is commonly affected by a thoracic aneurism or other tumour, for the same reasons we find, that if the apex of the right lung is diseased, the right recurrent laryngeal nerve will occasionally suffer, owing to the intimate relation between the right apex and the corresponding recurrent laryngeal nerve. I have never seen a case in which the left recurrent laryngeal nerve was affected as the result of pulmonary induration. Treatment of the throat-trouble is, of course, of no value.

4. *Phthisis Laryngea.*— In a case of pulmonary consumption, catarrhal larygnitis and anæmia must be regarded with suspicion and carefully watched, and we shall find a certain number of these cases gradually showing signs of deposition of tubercle, and they will drift into phthisis laryngea.

I have been struck with the rapidity with which throat consumption spreads. I saw a case of rapid pulmonary phthisis a short time ago, in which the patient complained of pain on swallowing, and I found the epiglottis swollen, turban-like, and evidently infiltrated with tubercle. In a day or two he became hoarse, and then I found congestion of the vocal cords and slight swelling of the mucous membrane over the arytenoids, especially over one of them. In a few days more the patient died, and the arytenoid swelling was found post mortem to be nearly as large as a hazel-nut.

I have also been struck with the small amount of deviation from the normal structure and appearance of the larynx, which is sufficient to cause a considerable degree of hoarseness; *e.g.*, I have examined patients who have had pulmonary phthisis in its early stages, with associated hoarseness, and I could only see some tiny out-growths between the arytenoids; the vocal cords and other parts appearing to be quite healthy.

In its early stages the treatment of phthisis laryngea ought not to be of an active and meddlesome character; and general advice as to avoiding changes of temperature, damp and cold atmosphere, hot and irritating food and drink, and tobacco smoking, combined with tonics and drugs by inhalation, &c., to relieve the cough, is of more importance than any local throat treatment.

When, however, ulceration of tubercular deposits has taken place, and there is much pain on taking food, along with hoarseness and irritating cough, then we must adopt systematic local treatment in the shape of spraying or brushing the affected parts with solutions of cocain in glycerine of borax, 5 to 10 per cent.; cocain pastilles; insufflations of iodoform or iodol; lactic acid carefully applied with the brush, and varying in strength from 20 to 60 or even 80 per cent., gradually increasing the concentration as the parts become tolerant of it. Solutions of menthol in olive oil to allay pain, and insufflations of morphia and bismuth for the same purpose, are good. All these measures must be combined with the usual cough mixtures, tonics, cod-liver oil, &c., which are used in cases of pulmonary phthisis.

The question of climate also is very important, and from what I have seen of patients in Davos and St. Moritz, having just returned from a visit to those places, I do not believe that we ought to send our phthisical cases there if the throat is seriously implicated, as the dry rarified air of the Engadine is very irritating even to a normal throat, until one gets acclimatized. Such patients ought to go to a warm climate, such as the Riviera, which, whilst I do not believe it is so valuable as that of the High Alps for the lung-trouble and for the general health, yet looking to the serious prospect of worrying a consumptive larynx, it is to be preferred.

5. *Coincidental Larnygeal Syphilis.*—This will necessarily occur in some phthisical patients, and must be treated *secundum artem.*

